# miR-19b targets pulmonary endothelial syndecan-1 following hemorrhagic shock

**DOI:** 10.1038/s41598-020-73021-3

**Published:** 2020-09-25

**Authors:** Feng Wu, Jian-Ying Wang, Wei Chao, Carrie Sims, Rosemary Ann Kozar

**Affiliations:** 1 Shock Trauma Center, University of Maryland School of Medicine, Baltimore, MD USA; 2 Cell Biology Group, Department of Surgery, University of Maryland School of Medicine, Baltimore, MD USA; 3 Baltimore Veterans Affairs Medical Center, Baltimore, MD USA; 4 Department of Pathology, University of Maryland School of Medicine, Baltimore, MD USA; 5 Department of Anesthesiology, University of Maryland School of Medicine, Baltimore, MD USA; 6 Division of Trauma, Critical Care and Burn, Ohio State University Wexner Medical Center, Columbus, OH USA

**Keywords:** Trauma, Translational research

## Abstract

Hemorrhagic shock results in systemic injury to the endothelium contributing to post-shock morbidity and mortality. The mechanism involves syndecan-1, the backbone of the endothelial glycocalyx. We have shown in a rodent model that lung syndecan-1 mRNA is reduced following hemorrhage, whereas the molecular mechanism underlying the mRNA reduction is not clear. In this study, we present evidence that miR-19b targets syndecan-1 mRNA to downregulate its expression. Our results demonstrate that miR-19b was increased in hemorrhagic shock patients and in-vitro specifically bound to syndecan-1 mRNA and caused its degradation. Further, hypoxia/reoxygenation (H/R), our in vitro hemorrhage model, increased miR-19b expression in human lung microvascular endothelial cells, leading to a decrease in syndecan-1 mRNA and protein. H/R insult and miR-19b mimic overexpression comparably exaggerated permeability and enhanced endothelial barrier breakdown. The detrimental role of miR-19b in inducing endothelial dysfunction was confirmed in vivo. Lungs from mice undergoing hemorrhagic shock exhibited a significant increase in miR-19b and a concomitant decrease in syndecan-1 mRNA. Pretreatment with miR-19b oligo inhibitor significantly decreased lung injury, inflammation, and permeability and improved hemodynamics. These findings suggest that inhibition of miR-19b may be a putative therapeutic avenue for mitigating post shock pulmonary endothelial dysfunction in hemorrhage shock.

## Introduction

Trauma is the leading cause of death in individuals up to age 46 and the third leading cause of death across all age groups^1,2^. Death from trauma occurs primarily from hemorrhage, brain injury, and multiple organ failure but hemorrhage remains the number one cause of early potentially preventable deaths^3^. Survivors of hemorrhagic shock develop an endotheliopathy of trauma which is a systemic response to activated endothelial cells leading to abnormalities in coagulation, inflammation, and endothelial barrier integrity^4^. Clinically, patients demonstrate a pro-inflammatory state as evidenced by a high incidence of systemic inflammatory response syndrome^5^. Endothelial hyperpermeability and subsequent tissue edema contributes to the development of multiple organ dysfunction and ultimately late deaths. The precise mechanism driving post-shock endothelial dysfunction, however, is not well known.

The endothelial glycocalyx is composed of proteoglycans and glycoproteins that project from endothelial cells to provide a protective layer^6,7^. Proteoglycans provide the structural support and consists primarily of syndecan to which the glycosaminoglycans attach. Sdc1 ectodomain is shed following a multitude of insults including hemorrhagic shock and sepsis^6,8–11^. Sdc1 ectodomain shedding results in loss of the endothelial glycocalyx and is regarded as a systemic biomarker for endothelial injury. We have been interested in the role of Sdc1 in endothelial dysfunction following hemorrhagic shock^6,8,12^. Our own and other studies demonstrated that Sdc1 loss is also associated with endothelial hyperpermeability, inflammation and shock, and is an independent predictor of mortality in patients^8,10,12^. The precise mechanism by which syndecan contributes to barrier integrity has not been well-elucidated. Our recent data demonstrated that fibrinogen (a major protein in blood) binding to sydnecan-1 on the endothelial cell surface activates the PAK1/cofilin intra-cellular signaling pathway to maintain endothelial barrier integrity^13,14^. However, much less is known about the regulation of Sdc1 expression, representing a gap in our knowledge. We have found in a rodent model of hemorrhagic shock that pulmonary Sdc1 mRNA is significantly reduced^8^, implicating that Sdc1 mRNA expression is also a target of hemorrhagic shock.

miRNAs are short single stranded RNA molecules that downregulate gene expression by binding with the 3′-untranslated regions (3′UTRs) of target mRNAs to inhibit translation and/or stability. Uhlich et al. recently identified 69 differentially expressed miRNAs in a small sample of severely injured trauma patients^15^. Based to these results, we searched three online miRNA-target prediction tools, TargetScan (https://www.targetscan.org), miRDB (https://www.mirdb.org) and miRanda (https://www.microrna.org) to determine if any of the 69 differentially modulated miRNAs target Sdc1. We identified miR-19b as the most likely effector for Sdc1 in injured patients. We therefore examined if miR-19b-induced downregulation of Sdc1 mRNA following hemorrhagic shock results in breakdown of endothelial cell barrier integrity and subsequent organ dysfunction.

## Materials and methods

### Human study

The human study was approved by the Institutional Review Board of the University of Pennsylvania School of Medicine. Samples were collected as part of recently completed interventional trial. Delayed informed consent was obtained from all patients and included the consent to investigate biologic markers of inflammation in the present study. Samples were de-identified and stored prior to bulk analysis^16^. All experimental procedures were conducted in compliance with the University of Maryland Baltimore and the National Institutes of Health guidelines on nucleic acid research. Human plasma samples were randomly obtained from 11 patients presenting in hemorrhagic shock at the time of arrival to the emergency room. For healthy donor controls, aliquots were obtained from 8 random donor units of fresh frozen plasmas obtained from Tennessee Blood Services (Memphis, TN). Plasma RNA was extracted using Trizol LS (Thermo Fisher scientific) and was reverse-transcribed (RT) using miScript II RT Kit (Qiagen). miR-19b quantative PCR (qPRC) analysis was performed using miScript SYBR Green PCR Kit (Qiagen) and miR-19b-3p miScript Primer (Qiagen). RNU6-2 miScript Primer (Qiagen) was used as an endogenous control. Relative RNA amount was calculated using the 2^-ΔΔCt method.

### In vitro

As lung is prone to injury after hemorrhagic shock^17^, we focused our research on pulmonary microvascular endothelial cells and pulmonary function.

#### Primary endothelial cell culture

Human lung microvascular endothelial cells (HLMEC; Lonza, MD) were cultured as we described previously^13^.

#### miR-19b mimic and miR-19b inhibitor transfection

HLMECs were transiently transfected by incubation with 100 nM miR-19b mimics (19bm, hsa-miR-19b-3p miScript miRNA Mimic; Qiagen), scrambled siRNA (scRNA, Allstars Negative Control siRNA; Qiagen), miR-19b oligo inhibitors (anti-19b, anti-hsa-miR-19b-3p miScript miRNA Inhibitor; Qiagen), or negative miRNA inhibitors (neRNA, miScript Inhibitor Negative Control; Qiagen) and lipofectamine 2000 (Thermo Fisher Scientific) in antibiotic-free Opti-MEM (Thermo Fisher Scientific) for 24 h. The medium was then changed to the growth medium, and the cells were cultured for another 48 h for assays.

#### Luciferase reporter assay

The full human 3′UTRs of Sdc1 (1325–3309; access # NM_001006946) and the mutated 3′UTRs of Sdc1 were inserted into pEZX-MT05 vectors by GeneCopoeia (Rockville, MD). Target site mutations (1837–1840 GCAC to CGTG) were also generated by GeneCopoeia using the PCR products with the appropriate primers containing point substitutions. The sequences were verified by DNA sequencing. HEK293T cells were co-transfected with 100 ng reporter constructs plus 100 nM miR-19b mimics or scRNA in 100 μl /well in 96-well plates. The activities of Gaussia Luciferase (Gluc) and Secreted Alkaline Phosphatase (SEAP) were detected 24 h later using a Dual Luminescence kit (GeneCopoeia) and Gluc activity was normalized to SEAP activity.

#### Western blotting

The blots were probed with anti-Sdc1 antibody (sc-12765, Santa Cruz Biotechnolgy) and anti-GAPDH antibody (PA1-987, Thermo Fisher scientific).

#### Hypoxia/reoxygenation (H/R)

H/R was conducted as described previously^18^. For normoxia, cells were cultured in DMEM containing 2% FBS in normoxia and 5% CO2. For H/R, cells were cultured in DMEM containing 2% FBS and no glucose overnight then in hypoxia (94% N2, 1% oxygen, and 5% CO2) for 6 h, and reoxygenation (i.e., normoxia) for another 3 h or 6 h.

#### Immunofluorescence assay

After transfection with 100 nM miR-19b mimics or scRNA, cells were subjected to normoxia or H/R then fixed in 4% paraformaldehyde. The cells were stained with anti-Sdc1 antibody or anti-VE-cadherin antibody (sc-9989, Santa Cruz Biotechnolgy). The fluorescence intensity was quantified using the free basic function of Quantity One software and reported as relative fluorescence units.

#### Endothelial barrier integrity

HLMECs were plated on gelatin-coated culture inserts (0.4 μm pore size; Corning Falcon) in 24-well companion plates and grown to confluence in EBM-2 containing 5% FBS and supplements. Some monolayers were transfected with 100 nM miR-19b mimics, scRNA, miR-19b oligo inhibitors, or neRNA and subjected to normoxia and H/R as described above. FITC-dextran transwell permeability assay and stress fiber staining were performed as we described previously^13^.

#### Biotin-conjugated miR-19b pulldown assays

Biotinylated miRCURY LNA miR-19b-3p mimics and biotinylated miRCURY LNA scrambled RNA were purchased from Qiagen. According to the product instruction, biotinylated miRCURY LNA miRNA Mimics consist of three RNA strands: a 3′ biotinylated miRNA (guide) strand with sequence exactly according to miRBase annotation and the passenger strand, which is split into two LNA-modified RNA strands complementary to the miRNA strand. Only the miRNA strand is incorporated by the RISC. The two passenger strands are too short to act as miRNAs and are rapidly degraded after displacement from the miRNA strand. Briefly, 100 nM biotin-conjugated miR-19b-3p or biotin-conjugated scRNA was transfected into HLMECs and whole-cell lysates were collected 48 h after transfection. The bead-bound RNAs were extracted with Trizol and subjected to RT-qPCR analysis as described previously^19^. RNA first strand was synthesized using SuperScript III First-Strand Synthesis System (Thermofisher Scientific) and quantative PCR was performed using iTaq Universal SYBR Green Supermix (Bio-Rad). The primer sequences used for detecting Sdc1 were: 5′-GAAGAAGAAGGACGAAGGCAG-3′ and 5′-CCTCCTGTTTGGTGGGC-3′. To determine the specificity of miR-19b binding to Sdc1 mRNAs, we also measured the possible targets of miR-19b (RORA:RAR related orphan receptor A and S1PR1:sphingosine-1-phosphate receptor 1) obtained by the online miRNA-target prediction tools described above as well as non-targets (TM: thrombomodulin and GPC1:glypican 1) with known structural similarity to Sdc1. The primer sequences were: RORA, 5′-GAGTTTGTGTTCTATGCACC-3′ and 5′-CCTTGCATATTAGCTTGGTTAG-3′; S1PR1, 5′-CATGAGGTGAAATGTGAGAG-3′ and 5′-AGTTGGTTGAAATGGATCAC-3′; TM, 5′-ACCTTCCTCAATGCCAGTCAG-3′ and 5′-GCCGTCGCCGTTCAGTAG-3′; and GPC1, 5′-AGCGACGTGGTCCGGAAAGT-3′ and CATGGAGTCCAGGAGGTTCCTCC-3′. GAPDH was used as an endogenous control. The primer sequences used for detecting GAPDH were: 5′-TGCACCACCAACTGCTTAGC-3′ and 5′-GGCATGGACTGTGGTCATGAG-3’.

#### mRNA stability assay

HLMECs were transfected with either 100 nM miR-19b mimics or scRNA. After 48 h, media was switched to media containing 10 μg/mL actinomycin D (Sigma-Aldrich) to inhibit transcription; each hour after that, total RNA was harvested using Trizol (Thermo Fisher scientific)^19^. Total RNA was isolated and the mRNA levels of Sdc1 and GAPDH were measured by RT-qPCR as described above.

### In vivo

#### Mouse model of hemorrhagic shock (HS)

All experimental procedures were approved by the Animal Care and Use Committee of Institutes of the University of Maryland School of Medicine and conducted in compliance with the National Institutes of Health guidelines on the use of laboratory animals. Adult male C57BL/6 J mice (9–12 weeks old) were subjected to our validated coagulopathic model of trauma-hemorrhagic shock^6^. Under isoflurane anesthesia, a midline laparotomy incision was made, the intestines were inspected and then the incision was closed. The right femoral artery was cannulated for continuous hemodynamic monitoring and blood withdrawal or resuscitation. Mean arterial blood pressure was recorded via the femoral arterial line at baseline then every 5 min during the shock period. After a 10-min period of equilibration, mice were bled to a mean arterial pressure (MAP) of 35 ± 5 mmHg for 90 min. Shams underwent anesthesia and placement of catheters but were not subjected to laparotomy or hemorrhagic shock. Mouse experiments were performed by repeating the order of sham, HS, HS + scRNA, and HS + miR-19b oligo inhibitor (HS + anti-19b) for randomization. Three hours after the end of shock, animals were sacrificed by exsanguination under isoflurane anesthesia and right lungs were harvested for RNA extraction and left lungs were filled with 0.4 ml 50% OCT in PBS then frozen for sectioning. A separate set of mice received Evans blue dye one hour before sacrificing for lung permeability assay^8^.

#### miR-19b oligo inhibitor treatment in vivo

Mice received 20 mg/kg subQ of LNA-stabilized miR-19b oligo inhibitor (5′-TGCATGGATTTGCAC-3′, synthesized by Exiqon for in vivo use) or LNA-stabilized scRNA control (synthesized by Exiqon for in vivo use) in 500 µl PBS following product instructions and the method description by Denby et al.^20^. Twenty-four hours after injection, mice in each group were subjected to trauma-hemorrhagic shock as described above.

#### Lung assays

Lung histopathology and myeloperoxidase (MPO) immunofluorescence staining were performed as we described previously^8,12^. Anti-MPO antibody (ab9535, Abcam) was used for the immunofluorescence staining. Lung permeability was measured by Evan’s blue dye extravasation assay^8^. miR-19b and Sdc1 mRNA were measured in the lung tissues by RT-qPCR. RNU6-2 and GAPDH were used as an endogenous control for miR-19b and Sdc1, respectively. Relative RNA amount was calculated using the 2^-ΔΔCt^ method (Supplementary File S1).

#### Statistical analysis

Data are expressed as mean ± SD. Values from different groups were analyzed by T test or one-way analysis of variance (ANOVA) with Bonferroni multiple comparison tests with significance set at level at p < 0.05.

## Results

### miR-19b is increased in hemorrhagic shock patients

**Figure 1:**
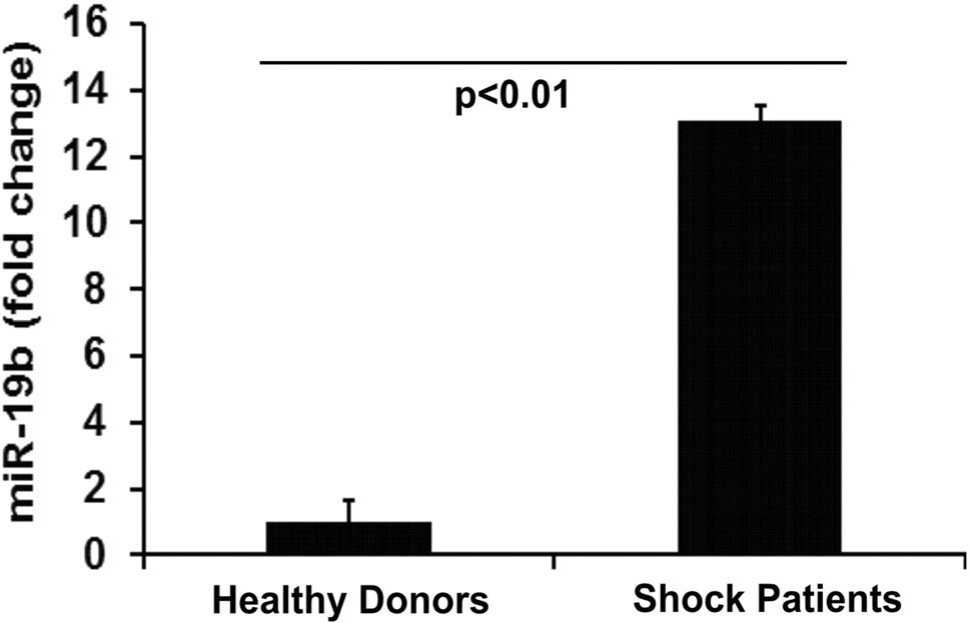
miR-19b is increased in human patients in hemorrhagic shock. Blood was obtained upon arrival in 11 injured patients presenting in shock (blood pressure < 90 mm Hg) who subsequently received blood administration as part of their initial resuscitation. Aliquots from 8 random donor units of fresh frozen plasma were used for controls. Shock patients had approximately a 13 fold increase in miR-19b. Data were expressed as mean ± SD.

miRNAs exist in stable form in the systemic circulation. Based on the expression profile of differentially regulated microRNAs identified by Uhlich et al. in hemorrhagic shock patients^15^, we performed quantitative analysis of miRNA-19b expression in a small cohort of patients with hemorrhagic shock. Consistent with the findings of Uhlich et al.^15^, miR-19b was significantly increased in the plasma of hemorrhagic shock patients (94% male) with a 13-fold increase compared to that of healthy donor controls (100% males) (Fig. 1).

### In vitro

#### H/R and miR-19b mimics comparably decreased Sdc1 and endothelial barrier integrity

**Figure 2:**
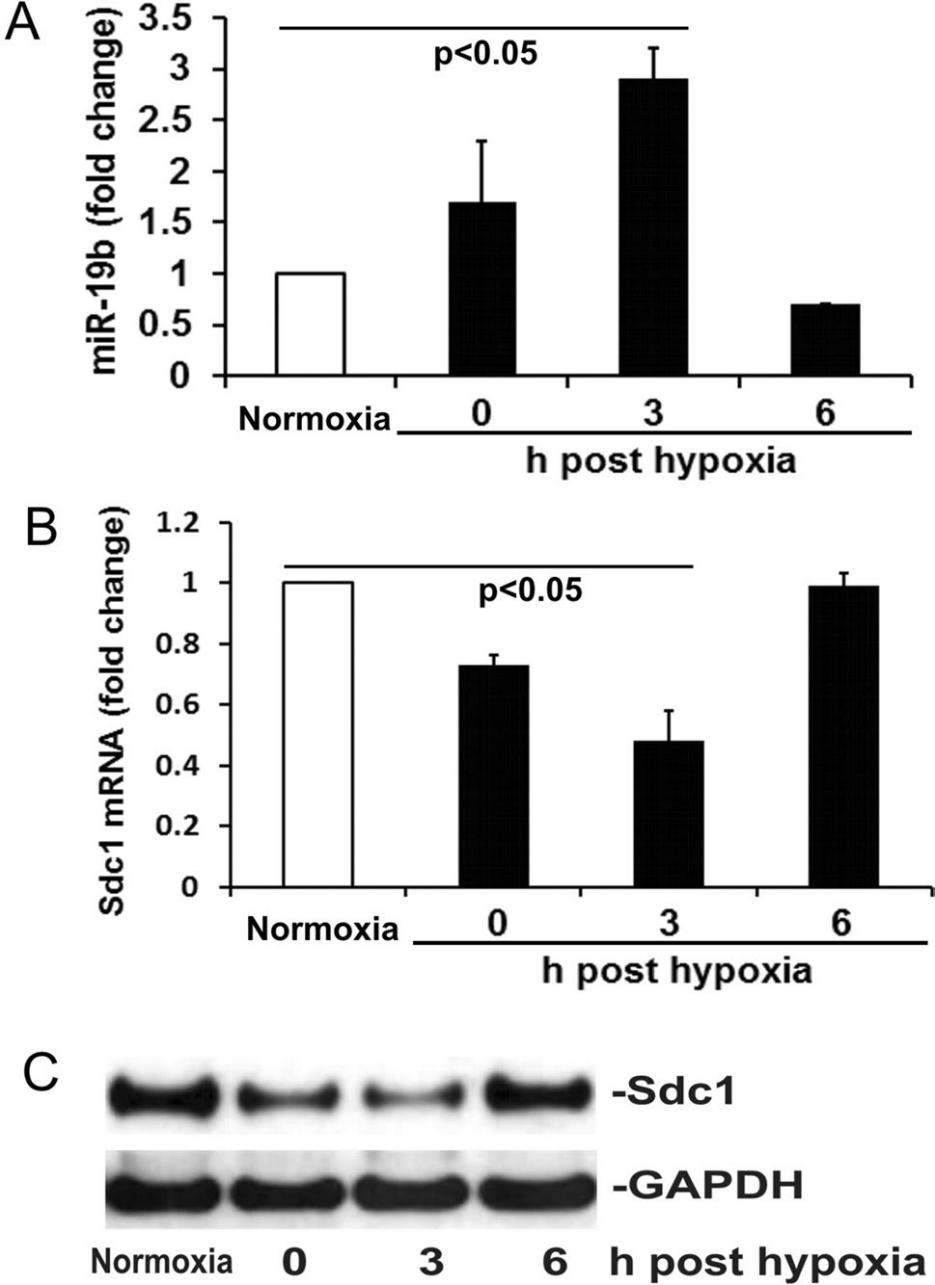
Hypoxia/reoxygenation (H/R) induces miR-19b expression and Sdc1 inhibition. (**A**,**B**) The levels of miR-19b and Sdc1 mRNA were measured by RT-qPCR in HLMECs exposed to either normoxia or hypoxia then reoxygenation at 0, 3 and 6 h. N = 4/group; mean ± SD; ANOVA with Bonferroni. (**C**) Expression of Sdc1 and GAPDH was detected by Western blot.

Using H/R as an in vitro model of hemorrhagic shock, we sought to investigate the contribution of miR-19b to endothelial dysfunction. Following exposure to H/R, HLMECs differentially expressed miR-19b and Sdc1, with a significant increase in miR-19b and a significant decrease in Sdc1 mRNA and protein at 3 h post hypoxia compared to normoxia only. At 6 h post hypoxia, both miR-19b and Sdc1 returned to normal levels (Fig. 2A–C).

**Figure 3:**
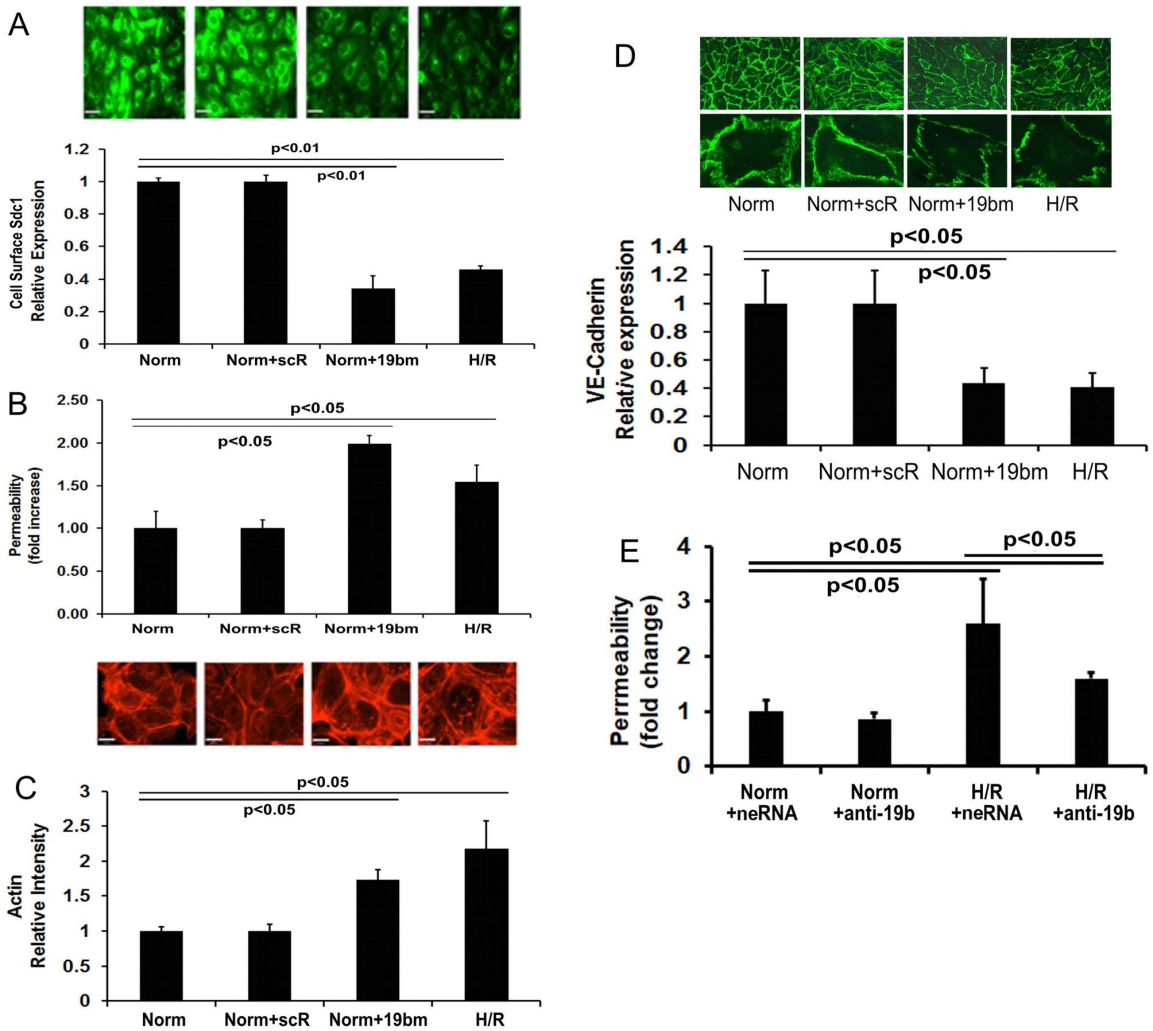
H/R or miR-19b mimics disrupts endothelial barrier but miR-19b oligo inhibitors prevent H/R-induced barrier disruption. In (**A**–**D**), HLMECs were exposed to normoxia (Norm), normoxia and transfected with scrambled RNA (Norm + scR), normoxia and transfected with miR-19b mimics (Norm + 19bm), or hypoxia/reoxygenation for 3 h (H/R). (**A**) Representative images of immunofluorescence-staining with anti-Sdc1 antibody were shown in the upper panel and relative Sdc1 fluorescent intensity quantitated using Quantity One software was reported in the lower panel. (**B**) Endothelial cell permeability to FITC-labeled dextran (40 kD) was measured and the fold change in FITC-dextran fluorescent intensity quantitated. (**C**) F-actin stress fibers were visualized using Texas red-X phalloidin. Representative images were shown in the upper panel and relative fluorescent intensity of fibers showed in the lower panel. (**D**) Representative images of immunofluorescence-staining with anti-VE-cadherin antibody were shown in the upper panel and relative VE-cadherin fluorescent intensity reported in the lower panel. In Fig. E, HLMECs were exposed to normoxia and transfected with either negative miRNA inhibitors (Norm + neRNA) or miR-19b oligo inhibitors (Norm + anti-19b), or exposed to hypoxia/reoxygenation for 3 h and transfected with either negative miRNA inhibitors (H/R + neRNA) or miR-19b oligo inhibitors (H/R + anti-19b). (**E**) Endothelial cell permeability to FITC-labeled dextran (40 kD) was measured and the fold change in FITC-dextran fluorescent intensity quantitated. N = 4/group; mean ± SD; ANOVA with Bonferroni. The images in this figure were analyzed using the free basic function of Quantity One 1-D Analysis Software (version #4.6.6). https://www.bio-rad.com/en-us/product/quantity-one-1-d-analysis-software?ID=1de9eb3a-1eb5-4edb-82d2-68b91bf360fb.

To further investigate the importance of the miR-19b/Sdc1 effector/target pair, Sdc1 cell surface expression was assessed after transfection with miR-19b mimics and demonstrated a reduction in expression, to an extent comparable to H/R (Fig. 3A). The ability of miR-19b mimics to phenocopy the reduction in Sdc1 expression induced by H/R suggests that miR-19b plays a key role in reducing Sdc1 after H/R. To investigate the functional consequences of miR-19b overexpression, endothelial barrier integrity was measured. Both miR-19b mimic overexpression and H/R insult comparably enhanced monolayer permeability, actin stress fiber formation and reduced expression of endothelial VE-cadherin when compared to scRNA and normoxia (Fig. 3B–D).

#### miR-19b selectively binds to Sdc1 mRNA and increases the degradation of Sdc1 mRNA

**Figure 4:**
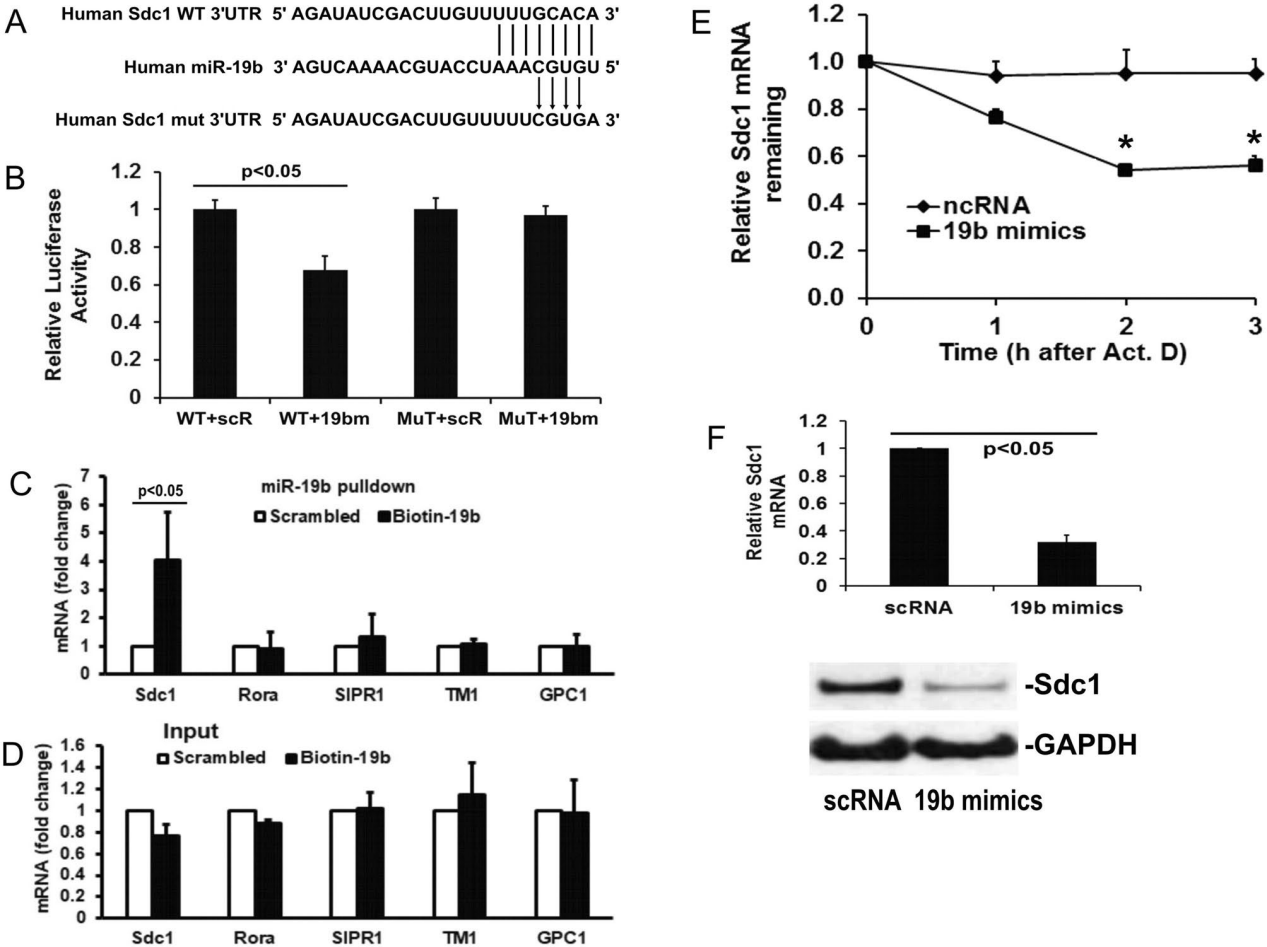
miR-19b selectively inhibits Sdc1. (**A**) Putative miR-19b target binding sites (1834–1841; access # NM_001006946) in human Sdc1 3′UTR as predicted by miRanda (https://www.microrna.org) and the mutated sites of Sdc1 3′UTR for luciferase activity assay. The figure shows alignment of miR-19b-3p with Sdc1 WT 3′UTR and the arrows indicate the mutagenesis nucleotides. (**B**) Relative luciferase activity in HEK293T cells co-transfected the reporter constructs containing wild type (WT) or mutant (mut) Sdc1 3′UTR with miRNA-19b mimics (19bm) or scrambled RNA (scR). (**C**) mRNA levels of Sdc1, RORA (RAR related orphan receptor A), S1PR1 (sphingosine-1-phosphate receptor 1), TM (thrombomodulin), and GPC1 (glypican 1) were measured in the mRNAs pulled with either biotin-conjugated miR-19b or biotin-conjugated scRNA. N = 3/group; mean ± SD; T test (**D**) mRNA levels of Sdc1, RORA, S1PR1, TM, and GPC1 were measured in the mRNAs isolated from the whole cell lysates. N = 3/group; mean ± SD. (**E**) HLMECs were transfected with either miR-19b mimics or scrambled RNA (scRNA) control. The remaining of Sdc1 mRNA was measured at 0, 1, 2 and 3 h after treatment with actinomycin D (Act. D, 10 µg/ml) in the cells. N = 4/group; mean ± SD; ANOVA with Bonferroni. (**F**) Sdc1 mRNA (upper panel) and protein (lower panel) in cells transfected with either scRNA or miR-19b mimics. GAPDH was detected as sample loading control.

Having established that miR-19b plays an important role in Sdc1 expression and function, we now sought to confirm that miR-19b targets and directly binds to Sdc1 mRNA. As shown in Fig. 4A, miR-19b demonstrates perfect 8-mer seed matches (1834–1841; access # NM_001006946) to Sdc1 3′UTR predicted by miRanda (https://www.microrna.org). To verify Sdc1 as a target gene of miR-19b, we performed a luciferase reporter gene assay. In the assay, when a miRNA is expressed and binds to the 3´ UTR, it results in repression of luciferase gene expression. Overexpression of miR-19b led to a reduction in luciferase activity in cells transfected with the 3′UTR of Sdc1 but had no effect when this binding site was mutated (Fig. 4B). Further, as shown in Fig. 4C,D, Sdc1 mRNA was markedly enriched in the mRNAs pulled with the biotin-conjugated miR-19b but not the mRNA pulled with biotin-conjugated scRNA. As a miRNA can inhibit a number of target mRNAs, we assessed other potential mRNA targets of miR-19b. In the pulldown mRNAs, we did not detect an increase in the possible targets of miR-19b, RORA and S1PR1, nor in the structurally similar non-targets, TM and GPC1. The biotin-conjugated miR-19b transfection had no significant effect on the total mRNA levels of Sdc1, RORA, S1PR1, TM, and GPC1. These results indicate that miR-19b binds specifically to Sdc1 mRNA and forms the miR-19b/Sdc1 mRNA complex. Furthermore, miR-19b mimic overexpression enhanced decay of Sdc1 mRNA leading to about ~ 50% drop of the original levels at 2 h after treatment with actinomycin D (Fig. 4E). Consistently, Sdc1 mRNA and protein decreased significantly in cells following transfection with miR-19b mimics compared to those cells transfected with scRNA (Fig. 4F). Notably, compared with miR-19b mimics used for functional study (Figs. 3A–D, 4F), the biotinylated-miR-19b has less efficiency in silencing Sdc1, as reported in other biotinylated-miRNA studies^21^. As expected, transfection with biotinylated-miR-19b for 48 h only slightly decreased the levels of Sdc1 mRNA (Fig. 4D). Whereas, Sdc1 expression was markedly inhibited in the cells transfected with miR-19b mimics for 72 h (Fig. 4F).

### In vivo

#### miR-19b oligo inhibitor protects Sdc1 expression in hemorrhage shock

**Figure 5:**
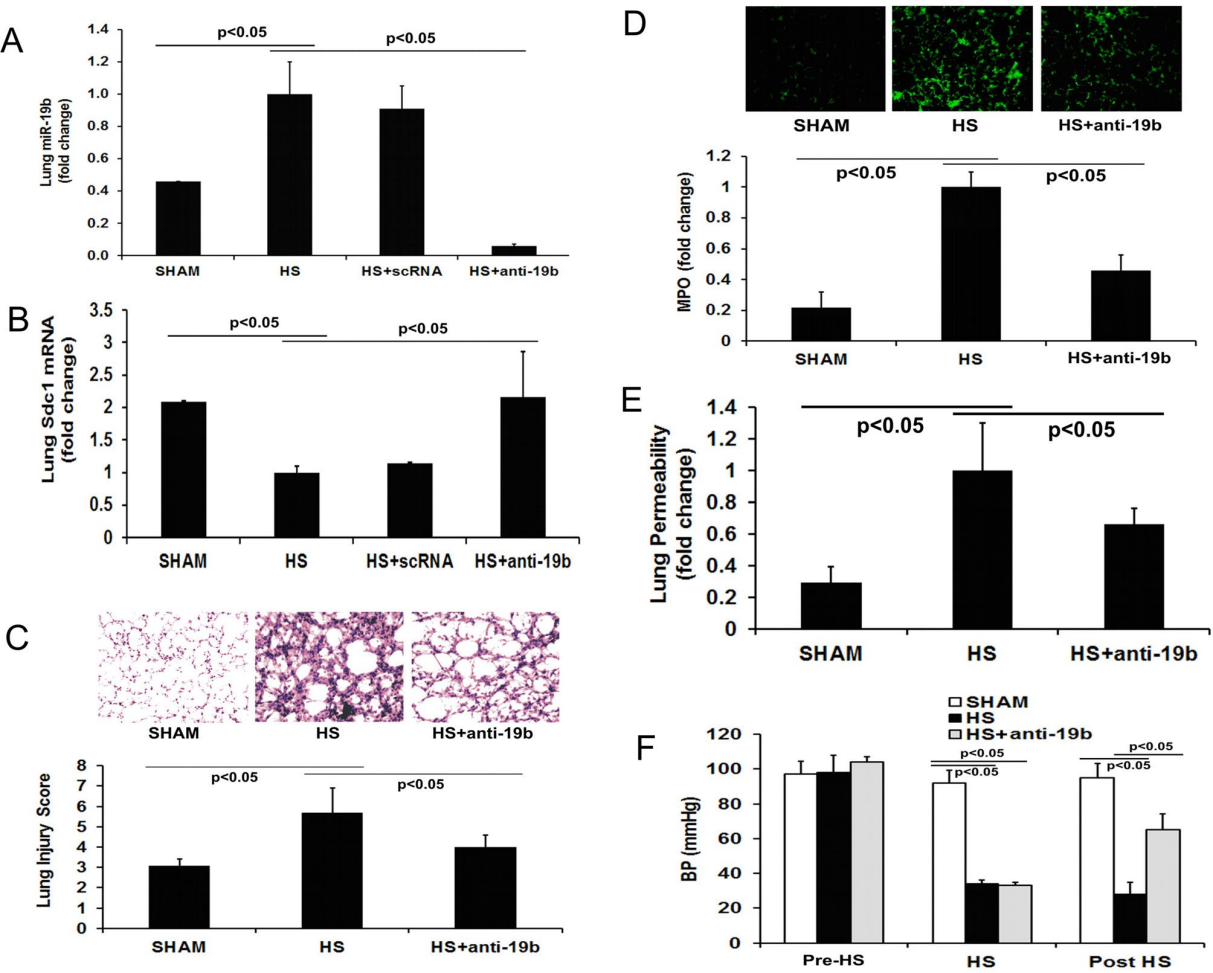
miR-19b oligo inhibitor mitigates lung inflammation and pulmonary vascular leakage and improves hemodynamics after hemorrhagic shock. Mice received 20 mg/kg subQ of miR-19b oligo inhibitor (anti-19b) or scrambled RNA (scRNA) then 24 h later underwent hemorrhagic shock (HS) and were sacrificed at 3 h. (**A**) miR-19b and (**B**) Sdc1 mRNA were measured by RT-qPCR in the lung tissues; there was approximately a 90% inhibition of pulmonary miR-19b by the oligo inhibitor which resulted in a return of pulmonary Sdc1 to sham levels after HS; n = 5/group. (**C**) Lung histopathology was assessed in tissue sections stained with H&E then scored using a 3-point scale each for alveolar thickness, capillary red cell retention and leukocyte infiltration. (**D**) Lung sections were immunostained for myeloperoxidase (MPO) and quantitated; representative images of MPO staining were shown in the upper panel and fold changes of relative fluorescence units in the lower panel. (**E**) Lung permeability was assessed using Evan’s blue dye extravasation assay. (**F**) Blood pressure recordings at pre-shock, during shock, and at 3 h post shock. N = 5/group; mean ± SD; ANOVA with Bonferroni. The images in this figure were analyzed using the free basic function of Quantity One 1-D Analysis Software (version #4.6.6). https://www.bio-rad.com/en-us/product/quantity-one-1-d-analysis-software?ID=1de9eb3a-1eb5-4edb-82d2-68b91bf360fb.

To assess the contribution of miR-19b to lung dysfunction following hemorrhagic shock, miR-19b oligo inhibitor was administered to mice prior to the induction of hemorrhagic shock. The oligo inhibitor successfully suppressed miR-19b expression in the shock lungs, leading to ~ 90% reduction compared to that in hemorrhagic shock alone (HS 1.00 ± 0.20 vs HS + anti-19b 0.06 ± 0.01 fold change). This reduction in miR-19b was accompanied by a corresponding increase in Sdc1 mRNA. Scrambled oligo inhibitor had no effect on miR-19b and Sdc1 mRNA in the shock lungs (Fig. 5A,B).

#### miR-19b oligo inhibitor reduced lung dysfunction in hemorrhage shock

Consistently, miR-19b oligo inhibitor also reduced post-shock lung histopathologic injury, inflammation, and permeability, compared to those in shock alone animals (Fig. 5C–E). Moreover, the oligo inhibitor had a significant effect in improving systemic hemodynamics. Despite similar pre-shock blood pressure and a similar degree of shock, at 3 h post shock, the oligo inhibitor-treated mice had a significantly higher blood pressure compared to mice not receiving the inhibitor (HS + anti-19b: 65 ± 4.0 vs HS: 28 ± 3.5 mm Hg) (Fig. 5F).

## Discussion

We identified for the first time that miR-19b targets Sdc1. In our small proof-of-concept study in trauma patients, we confirmed that miR-19b was significantly increased in hemorrhagic shock patients. Using H/R as an in vitro model of hemorrhage shock, our results indicated that pulmonary endothelial cells differentially expressed miR-19b and Sdc1. Overexpression of miR-19b mimics phenocopied the reduction in Sdc1 expression and the increase in permeability observed in endothelial cells after H/R. MiR-19b biotin-based pulldown assay confirmed that miR-19b specifically binds to Sdc1 and mRNA stability assay revealed that miR-19b degrades Sdc1 mRNA. Endothelial dysfunction induced by overexpression of miR-19b mimics in vitro was confirmed in vivo by pretreatment of hemorrhagic shock mice with miR-19b oligo inhibitor, which maintained Sdc1 expression and attenuated post-shock lung injury, inflammation and hyperpermeability.

There is only one reported miRNA study in trauma patients. We validated the differential expression of miR-19b first identified by Uhlich et al.^15^ in hemorrhagic shock patients and reported for the first time that miR-19b targets Sdc1, providing additional mechanistic insight underlying the loss of endothelial Sdc1 after hemorrhage shock. We and others have found a marked increase in shedding of the Sdc1 ectodomain in patients in hemorrhagic shock^6,11,12,22–24^. Sdc1 ectodomains are cleaved at the plasma membrane and considered biomarkers of endothelial injury and independent predictors of mortality^10–12,25^. Much less is known regarding the regulation of endothelial Sdc1 mRNA in hemorrhage shock but our present results demonstrated a significant reduction in Sdc1 mRNA expression that is mediated by miR-19b. Importantly, a decrease in Sdc1 mRNA is associated with endothelial dysfunction, a hallmark of the endotheliopathy of trauma^12,26^.

miR-19b belongs to the miR-17–92 family of miRNA clusters. It has been shown to play a role in several cancers^27,28^ and to be associated with inflammatory conditions including atherosclerosis^28,30^. It has also been linked specifically to endothelial cell dysfunction in the progression of atherosclerosis^31^. Li et al. demonstrated elevated systemic levels of miR-19b in patients with unstable angina and coronary artery disease^32^. Interestingly, they found that miR-19b is primarily found in circulating endothelial microparticles^32^. In our small proof-of-concept study in injured patients, we were able to confirm that systemic miR-19b was significantly increased in hemorrhagic shock patients. However, we did not have sufficient plasma samples to determine if miR-19b is incorporated into microparticles. A prospective study is in progress to answer this question. More recently, Li et al. determined that miR-19b containing microparticles promoted secretion of pro-inflammatory cytokines in peri-endothelial tissue^33^. Whether miR-19b promotes the known pro-inflammatory cytokine release from endothelial cells following hemorrhagic shock is unknown.

miR-19b has been shown to play a role in a LPS model of acute kidney injury^34^. Lv et al. demonstrated an increase in miR-19b in renal exosomes. These exosomal miR-19b targeted SOCS1 (suppressor of cytokine signaling 1) in macrophages and resulted in M1 phenotypes. Moreover, injection of exosomal miR-19b to mice caused renal tubulointerstitial inflammation^34^. These results support that miR-19b is a pro-inflammatory miRNA.

Although we demonstrated that miR-19b targets Sdc1, all miRNAs have many potential targets. miR-19b can target genes other than Sdc1 and may do so after hemorrhagic shock. However, our results support that miR-19b selectively targets Sdc1 mRNA after hemorrhage. Biotin-based pulldown assay demonstrated binding of miR-19b to Sdc-1 but not to other possible targets or non-targets with structural similarity to Sdc1.

In conclusion, this study demonstrates Sdc1 to be a novel target of endothelial cell miR-19b after hemorrhagic shock. Inhibition of endothelial miRNA-19b may be a putative therapeutic avenue for mitigating post shock endothelial dysfunction.

## Supplementary information

Supplementary file 1
