# Supplementary figures and images for: miR-19b targets pulmonary endothelial syndecan-1 following hemorrhagic shock
Source: Sci Rep. 2020 Sep 25;10:15811. doi: 10.1038/s41598-020-73021-3 (PMC7519668; doi:10.1038/s41598-020-73021-3)

Fig. 2C

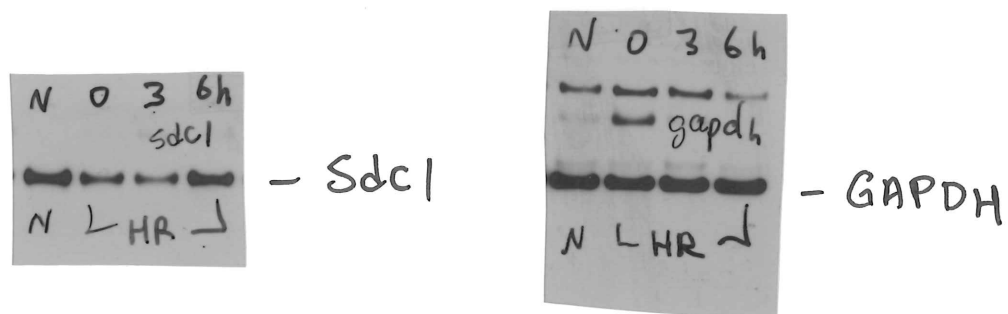

Fig. 4F

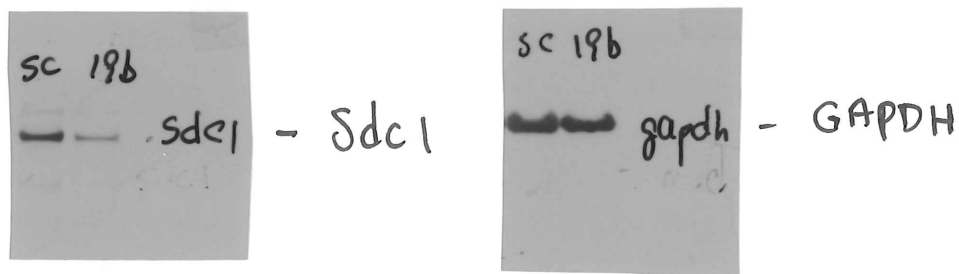

Supplement: Supplementary file 1 [file 41598_2020_73021_MOESM1_ESM.pdf]
